# Differential expression of a virulence factor in pathogenic and non-pathogenic mycobacteria

**DOI:** 10.1111/j.1365-2958.2009.06612.x

**Published:** 2009-02-19

**Authors:** Edith N G Houben, Anne Walburger, Giorgio Ferrari, Liem Nguyen, Charles J Thompson, Christian Miess, Guido Vogel, Bernd Mueller, Jean Pieters

**Affiliations:** 1 Biozentrum, University of BaselKlingelbergstrasse 50/70, 4056 Basel, Switzerland; 2 Hoffmann-La Roche Ltd.Grenzacherstrasse 124, 4070 Basel, Switzerland; 3 Kantonales Laboratorium Basel-StadtKannenfeldstrasse 2, 4012 Basel

## Abstract

The pathogenicity of mycobacterial infections depends on virulence factors that mediate survival inside host macrophages. These virulence factors are generally believed to be specific for pathogenic species and absent or mutated in non-pathogenic strains. The serine/threonine protein kinase G (PknG) mediates survival of mycobacteria within macrophages by blocking lysosomal delivery. Here we describe a gene of the non-pathogenic species *Mycobacterium smegmatis* that is 78% identical with *pknG* of *Mycobacterium tuberculosis* and *M. bovis* bacillus Calmette–Guérin (BCG). When cloned into expression vectors, the *M. smegmatis pknG* orthologue produced an active kinase and performed the same function as its *M. bovis* BCG counterpart in intracellular survival. In addition, similar levels of *pknG* transcripts were found in *M. bovis* BCG and *M. smegmatis*. However, virtually no translation product of chromosomal *pknG* could be detected in *M. smegmatis* both after *in vitro* growth and after macrophage infection. This lack of efficient translation was shown to be caused by regulatory elements in the upstream region of the *M. smegmatis* gene. The data reveal dramatically increased translational efficiency of a virulence gene in a pathogenic mycobacterium compared with a non-pathogenic mycobacterium suggesting that changes in expression levels may underlie evolution of *pknG* and other pathogenicity genes in mycobacterium.

## Introduction

Pathogenic mycobacteria, such as *Mycobacterium tuberculosis*, utilize an array of different strategies to survive inside mammalian host cells. Survival strategies are employed during the initial phases of the infection when the bacteria acquire access to their host cells, as well as during later stages of infection as the bacteria convert into a so-called dormant or latent state (Cosma *et al*., 2003). In the initial phases of infection, it is important that mycobacteria gain entry to macrophages, as they have the unique capacity to avoid antibacterial effectors of the immune system in this intracellular niche (Russell, 2001; Vergne *et al*., 2004; Houben *et al*., 2006). To ensure survival inside macrophages, pathogenic mycobacteria avoid their degradation by actively inhibiting fusion of the mycobacterial phagosome with lysosomes (Armstrong and Hart, 1971; Russell, 2001; Houben *et al*., 2006).

The molecular mechanisms that are responsible for diverting the traffic of pathogenic mycobacteria from the lysosomal degradative pathway are beginning to be understood and both host factors and mycobacterial molecules are involved in this process (Vergne *et al*., 2004; Houben *et al*., 2006). The eukaryotic-like serine/threonine kinase, protein kinase G (PknG), is one of the mycobacterial factors involved in blocking lysosomal delivery (Walburger *et al*., 2004; Scherr *et al*., 2007). Although it has been reported that PknG in *M. tuberculosis* and a related microorganism (*Corynebacterium glutamicum*) is required for optimal *in vitro* growth and is linked to intracellular glutamine/glutamate levels (Cowley *et al*., 2004; Niebisch *et al*., 2006), PknG in *Mycobacterium bovis* bacillus Calmette–Guérin (BCG) has been shown not to be required for mycobacterial growth outside macrophages in a complete liquid medium and not to be involved in glutamine metabolism (Walburger *et al*., 2004; Nguyen *et al*., 2005). After their uptake within macrophage phagosomes however, pathogenic mycobacteria lacking PknG are readily transported to lysosomes and destroyed (Walburger *et al*., 2004 and data not shown).

Interestingly, also the non-pathogenic *Mycobacterium smegmatis* genome contains a gene that is very similar to *M. tuberculosis* and *M. bovis pknG*. We here characterize the gene product from *M. smegmatis pknG*, showing that it encodes an active serine/threonine kinase and is able to block the lysosomal delivery of mycobacteria inside macrophages when expressed from expression vectors. However, while PknG transcripts were found in *M. smegmatis*, chromosomal *pknG* was not effectively translated in this non-pathogenic mycobacterium. The data show that PknG expression is blocked on a translational level in *M. smegmatis* and suggest that translational efficiency is an important factor in assessing candidate virulence genes.

## Results

### Mycobacterial PknG orthologues

**Fig. 1:**
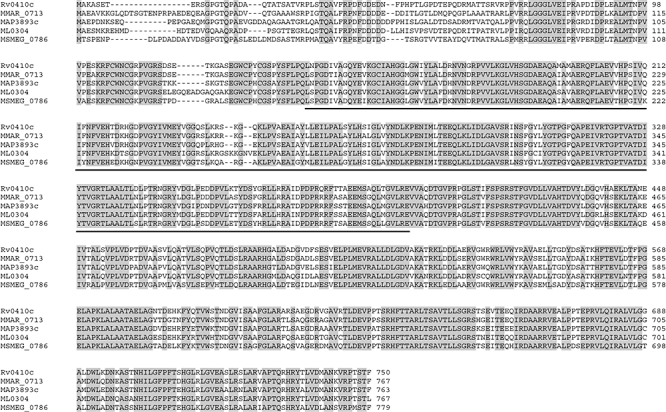
Alignment of mycobacterial protein kinase G orthologues. PknG sequences from *M. tuberculosis* H37Rv (Rv0410c, from the Pasteur Institute), *M. marinum* (MMAR_0713, from the Sanger Institute), *M. avium paratuberculosis* (MAP3893c, from TIGR), *M. leprae* (ML0304, from the Sanger Institute) and *M. smegmatis* (MSMEG_0786, from TIGR) were aligned using clustalw. The PknG orthologues of *M. bovis* and *M. microti* were omitted since they were identical to *M. tuberculosis* PknG. The *M. smegmatis* PknG coding sequence starts at the ATG that aligns to the start codon of *M. tuberculosis* PknG (see Fig. S1 online). Identical residues are shown in grey and the kinase domain is underlined.

**Fig. 8:**
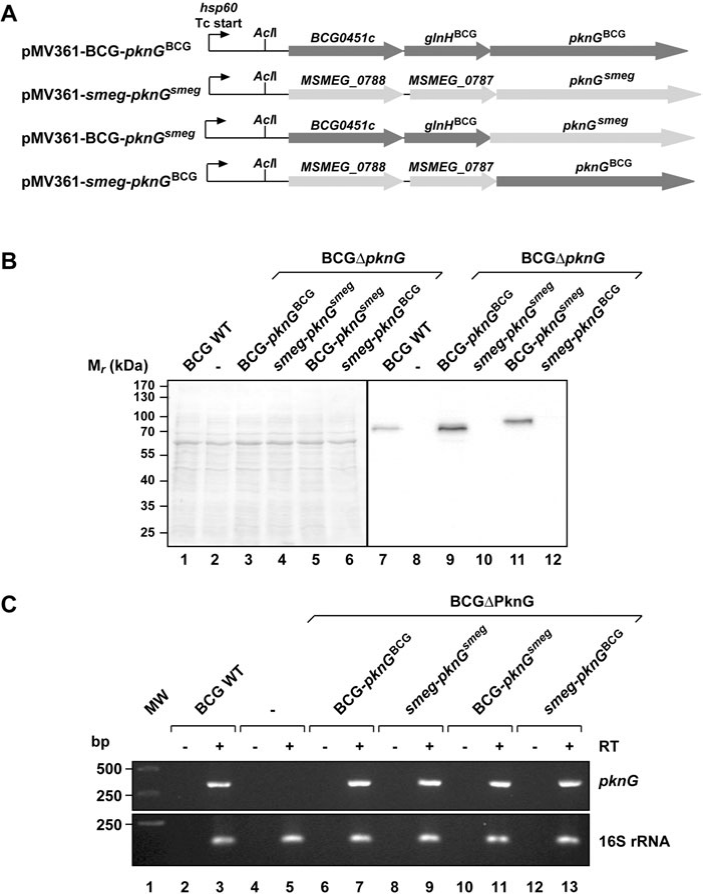
Analysis of the influence of the upstream region of *M. smegmatis* and *M. bovis* BCG *pknG* on *pknG* expression. A. Schematic representation of the fusion constructs used in this experiment. All constructs were introduced in the pMV361 vector at the AclI restriction site in the *hsp60* promoter leaving the transcription (Tc) start intact (see also *Experimental Procedures*). pMV361-BCG-PknG^BCG^, pMV361 with *M. bovis* BCG *pknG* and its own upstream region; pMV361-*smeg*-PknG^*smeg*^, pMV361 with *M. smegmatis pknG* and its own upstream region; pMV361-BCG-PknG^*smeg*^, pMV361 with *pknG*^*smeg*^ fused to the *M. bovis* BCG *pknG* upstream region; pMV361-*smeg*-PknG^BCG^, pMV361 with *pknG*^BCG^ fused to the *M. smegmatis pknG* upstream region. B. Lysates from *M. bovis* BCG (lane 7) and *M. bovis* BCGDΔ*pknG* transformed with empty vector (lane 8*)* or the plasmids shown under (A) (lanes 9–12) were separated on a 10% SDS-PAGE gel and immunoblotted using anti-PknG^BCG^ antiserum (right). The total protein pattern was analysed by Ponceau Red staining (left). C. cDNA was prepared from total RNA isolated from *M. bovis* BCG wt and the *M. bovis* BCGΔ*pknG* transformants analysed under (B) by using reverse transcriptase and random primers. cDNA of *pknG* was subsequently amplified using gene-specific primers. As controls the same reactions were carried out without reverse transcriptase (−RT) or in stead of *pknG* primers with 16S rRNA-specific primers. Representative data from three experiments are shown.

The mycobacterial serine/threonine kinase PknG is required for survival of pathogenic mycobacteria inside macrophages (Walburger *et al*., 2004). To analyse whether the presence of PknG is specific for pathogenic mycobacteria, all available mycobacterial genome sequences, i.e. of *M. tuberculosis*, *M. bovis* (BCG), *M. microti*, *M. marinum*, *M. avium* (*paratuberculosis*), *M. leprae* and *M. smegmatis*, were analysed for the presence of a locus encoding *pknG* orthologues (Fig. 1). All of these genomes encoded an open reading frame that was highly homologous to PknG of *M. tuberculosis*. Strikingly, the genome of the non-pathogenic *M. smegmatis* also encodes a *pknG* orthologue (named MSMEG_0786) showing 78% identity and 87% similarity to *M. tuberculosis* PknG. In addition, the postulated operon structure around the *pknG* gene is conserved in *M. smegmatis* as well (see also Fig. 8A). For all PknGs, the homology extends over the kinase domain (residues 141–398 of *M. tuberculosis* PknG) as well as the C-terminal region, with the only deviation from homology within the N-terminus. On a phylogenetic perspective, we conclude that the *pknG* gene was present in mycobacteria before the divergence of the fast-growing mycobacterial species (*M. smegmatis*), from the slow-growing species (pathogenic mycobacteria) (Shinnick and Good, 1994).

### Kinase activity of PknG from *M. smegmatis*

**Fig. 2:**
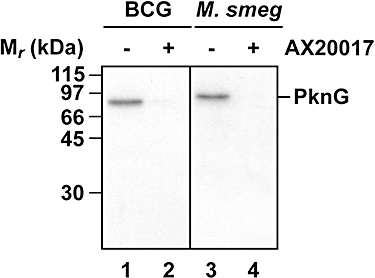
Kinase activity of PknG. The kinase activity of PknG was determined by incubation of 0.1 μg of purified *M. bovis* BCG and *M. smegmatis* His-PknG in the absence or presence of the specific PknG inhibitor AX20017 (1 mM) for 30 min at 37°C in a kinase buffer (25 mM Tris/HCl pH 7.4, 2 mM MnCl_2_, 1 mM DTT and 3.7 × 10^5^ Bq [γ-^32^P]-ATP). The reactions were terminated by the addition of SDS sample buffer and proteins were separated by 10% SDS-PAGE, fixed, dried and autoradiographed to determine the phosphorylation of the PknGs. Results are representative data from one experiment.

In several non- or low-virulent bacteria, virulence genes have been lost through deletion or acquisition of mutations that either prevent the expression of a full-length protein or give rise to an inactive product (Denecker *et al*., 2002; Higashi *et al*., 2002; Roche *et al*., 2005). To determine whether the *M. smegmatis* open reading frame encodes a functional kinase, as is predicted by its sequence, His-tagged versions of *M. smegmatis* PknG as well as *M. bovis* BCG PknG, which is identical to *M. tuberculosis* PknG (Walburger *et al*., 2004), were expressed in *Escherichia coli*, purified and their kinase activity was analysed. As shown in Fig. 2, *M. smegmatis* PknG was phosphorylated to a similar degree as *M. bovis* BCG PknG. Furthermore, inclusion of the PknG specific inhibitor AX20017 (Walburger *et al*., 2004; Scherr *et al*., 2007) blocked autophosphorylation of both PknGs. We conclude that the *M. smegmatis pknG* gene encodes a orthologue of *M. bovis* BCG PknG that is an enzymatically active serine/threonine kinase.

### *In vivo* function of *M. smegmatis* PknG

**Fig. 3:**
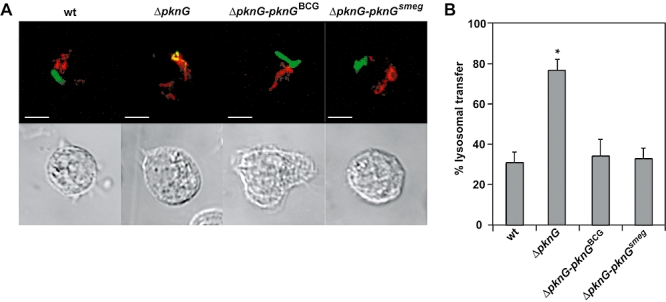
Subcellular localization of *M. bovis* BCG in the absence or presence of PknG. A. Bone marrow-derived macrophages were infected with *M. bovis* BCG (wt), *M. bovis* BCGΔ*pknG* (Δ*pknG*), *M. bovis* BCGΔ*pknG-*pMV361*-pknG*^BCG^ (Δ*pknG-pknG*^BCG^) or *M. bovis* BCGΔ*pknG-*pMV361*-pknG*^*smeg*^ (Δ*pknG-pknG*^*smeg*^) for 1 h, followed by a 2 h chase, fixed, permeabilized, and immunodecorated with antibodies raised against LAMP1 (rat) and *M. bovis* (rabbit). Anti-rat Alexa Fluor 568 (red) and anti-rabbit Alexa Fluor 488 (green) were used as secondary antibodies. Scale bar, 5 μm. B. For quantifications, cells containing mycobacteria in LAMP-positive vacuoles were scored. Results are mean values (± SD) from three independent experiments (50 mycobacteria containing vacuoles were scored per experiment). Compared with *M. bovis* BCG (wt) only non-complemented *M. bovis* BCGΔ*pknG* (Δ*pknG*) colocalized significantly more with LAMP1 (*P < 0.05).

We then investigated whether *M. smegmatis* PknG was able to carry out the same *in vivo* function as PknG of pathogenic mycobacteria, i.e. in blocking the fusion of mycobacterial phagosomes with lysosomes. For this, a PknG knockout of *M. bovis* BCG was transformed with the plasmids pMV361*-pknG*^*smeg*^ or pMV361*-pknG*^BCG^, in which PknG expression was directed by the mycobacterial *hsp60* promoter and translational initiation sequences. The intracellular trafficking of the transformants was subsequently analysed after infection of bone marrow-derived macrophages (Fig. 3). As observed previously, *M. bovis* BCGΔ*pknG* colocalized predominantly with the lysosomal marker LAMP1, while wild-type mycobacteria largely remained in LAMP1-negative vacuoles (Walburger *et al*., 2004). Importantly, this defect of the mutant was complemented with either pMV361*-pknG*^BCG^ (Houben *et al*., 2006) or pMV361*-pknG*^*smeg*^. We conclude that PknG from *M. smegmatis* is not only an enzymatically active serine/threonine kinase, but is also able to carry out the same biological function as PknG of *M. bovis* BCG in blocking lysosomal delivery of mycobacteria.

### Expression of *M. smegmatis* PknG during *in vitro* growth

**Fig. 4:**
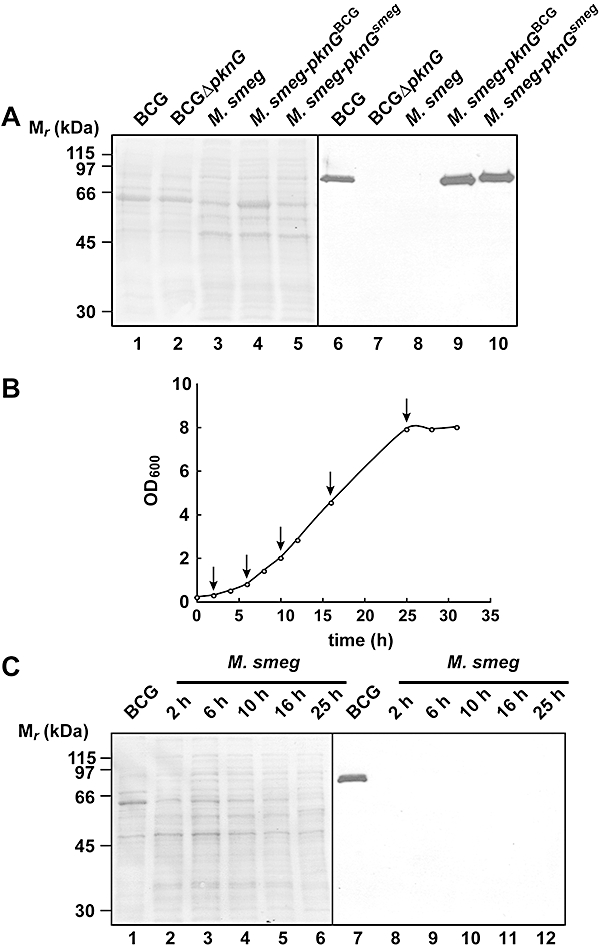
Expression of *M. smegmatis* PknG during *in vitro* growth. A. Lysates from *M. bovis* BCG (lane 1), *M. bovis* BCGΔ*pknG* (lane 2), *M. smegmatis* (lane 3), *M. smegmatis-pknG*^BCG^ (lane 4), *M. smegmatis-pknG*^*smeg*^ (lane 5) were separated on a 10% SDS-PAGE gel and immunoblotted using anti-PknG^BCG^ antiserum (right). The total protein pattern was analysed by Ponceau Red staining (left). B and C. *M. smegmatis* was grown in 7H9-OADC medium until stationary phase. Bacterial samples were collected at different time points (arrows in B) and analysed for the presence of PknG by immunoblotting as under A (C). BCG lysate was loaded in parallel as a positive control (lane 1). Again, the total protein pattern was analysed by Ponceau Red staining (left). Results are representative data from one experiment.

Initial experiments to investigate the expression of chromosomal *M. smegmatis pknG* by immunoblotting using antibodies against *M. bovis* BCG did not detect any endogenous PknG in *M. smegmatis* (Walburger *et al*., 2004). To determine whether this represented specificity of the antibody for *M. bovis* BCG PknG, *M. smegmatis* was transformed with the plasmids pMV361*-pknG*^*smeg*^ or pMV361*-pknG*^BCG^. Exponentially growing cultures of these *M. smegmatis* transformants and *M. bovis* BCG cultures were analysed by Western blotting using an antiserum raised against PknG^BCG^ (Fig. 4A). PknG^*smeg*^ expressed in *M. smegmatis* from pMV361 was readily recognized by the PknG antibodies (lane 10), to a similar degree as PknG from *M. bovis* BCG expressed endogenously (lane 6) or in *M. smegmatis* from pMV361*-pknG*^BCG^ (lane 9). Furthermore, recombinant PknG from *M. bovis* BCG and *M. smegmatis* that was produced in *E. coli* and purified generated comparable signals after immunoblotting (not shown). Therefore, the failure to detect PknG in *M. smegmatis* was not due to a defect in the cross-reactivity of the PknG^BCG^-specific antisera.

To explore the possibility that expression of PknG in *M. smegmatis* was growth phase dependent, an overnight culture of *M. smegmatis* was diluted to OD_600_ 0.1 and grown until the culture reached stationary phase (Fig. 4B). Samples were taken for immunoblotting of bacterial lysates at different time points during growth. As seen in Fig. 4C, no PknG could be detected using anti-PknG^BCG^ at any stage during growth of *M. smegmatis*, showing that PknG expression in *M. smegmatis* is not induced at a particular growth phase.

### Expression of *M. smegmatis* PknG after macrophage infection

**Fig. 5:**
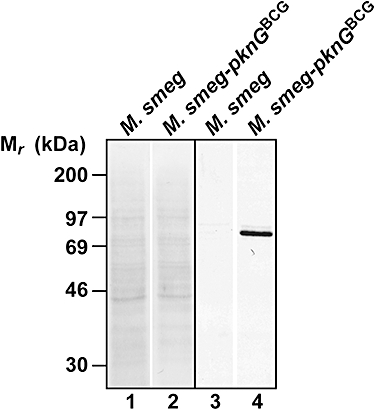
Expression of *M. smegmatis* PknG during macrophage infection. PknG immunoblot of macrophages infected for 16 h with *M. smegmatis* or *M. smegmatis* expressing PknG^BCG^. After infection, cells were harvested, homogenized and a post-nuclear supernatant was submitted to immunoblotting (lanes 3 and 4). The total protein pattern was analysed by Ponceau Red staining (lanes 1 and 2). Results are representative data from one experiment.

Although PknG is not detected in *M. smegmatis* growing *in vitro*, it is possible that expression is upregulated when *M. smegmatis* is phagocytosed by macrophages. To analyse this, bone marrow-derived macrophages were infected with either *M. smegmatis* wild type or *M. smegmatis*-expressing PknG^BCG^ from pMV361. After 16 h of infection, cells were harvested, homogenized and a post-nuclear supernatant was prepared and separated by SDS-PAGE followed by immunoblotting. As can be seen in Fig. 5, while PknG was readily observed in macrophages that had been infected with *M. smegmatis* expressing PknG^BCG^ controlled by the *hsp60* promoter, no PknG was detected in cells that had phagocytosed *M. smegmatis*. We therefore conclude that PknG is not upregulated in *M. smegmatis* upon infection of macrophages.

### Selected ion monitoring analysis of total *M. smegmatis* lysates

**Fig. 6:**
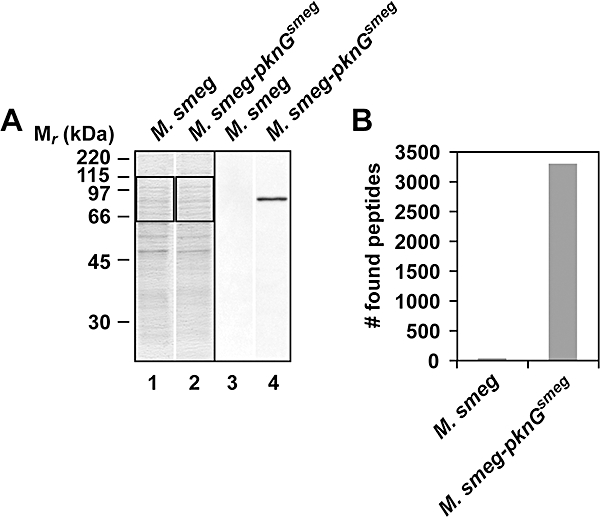
Selected ion monitoring analysis of *M. smegmatis* lysates. A. Lysates (10 μg) from *M. smegmatis* and *M. smegmatis-pknG*^*smeg*^ were separated on a 10% SDS-PAGE gel, followed by Coomassie Blue staining (lanes 1 and 2) or immunoblotting using anti-PknG^BCG^ antiserum (lanes 3 and 4). B. Thirty micrograms of the same lysates as analysed under (A) were separated on a 10% SDS-PAGE gel and stained with Colloidal Blue. The region between 65 and 115 kDa was excised (indicated by the rectangles in A), digested with trypsin and the obtained peptides were identified using selected ion monitoring (see *Experimental procedures*). Representative data from two independent experiments are shown.

Finally, a more sensitive mass spectrometry-based approach was adopted to detect PknG in *M. smegmatis*. Selected ion monitoring analysis (SIM) of defined peptide masses was employed to analyse the presence of PknG-derived peptides in *M. smegmatis* lysates. Total proteins from *M. smegmatis* and *M. smegmatis* transformed with pMV361*-pknG*^*smeg*^ were separated by SDS-PAGE. Proteins between 65 and 115 kDa were excised from the gel (Fig. 6A), subjected to protease digestion and analysed by mass spectrometry. Selected ion monitoring allowed the detection of two selected peptides 51 times in the wild-type *M. smegmatis* sample, while six peptides (see *Experimental procedures*) were identified 3289 times in a lysate from *M. smegmatis* expressing PknG^*smeg*^*in trans* (Fig. 6B). These data, together with the absence of a PknG signal following immunoblotting, show that PknG is translated at extremely low levels in *M. smegmatis*.

### Transcription of *pknG* from *M. smegmatis*

**Fig. 7:**
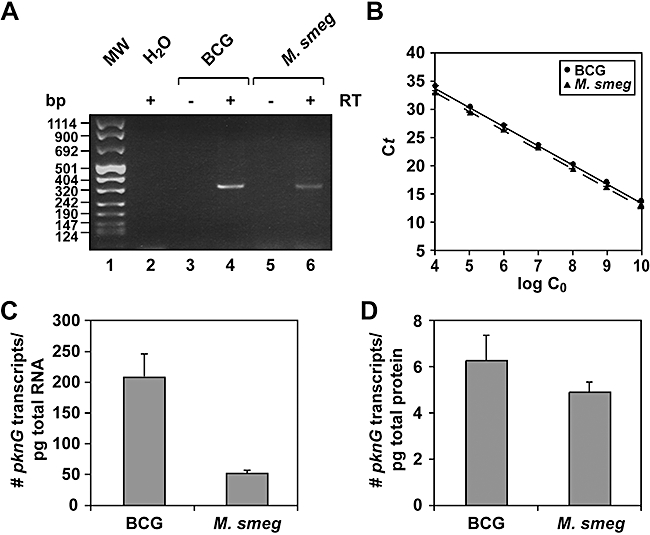
Analysis of *pknG* transcription in *M. smegmatis*. A. cDNA was prepared from total *M. bovis* BCG and *M. smegmatis* RNA by using reverse transcriptase and random primers. cDNA of *pknG* was amplified using gene-specific primers. As a control the same reactions were carried out without reverse transcriptase (−RT) or without RNA (H_2_O). B. Standard curves for *M. bovis* BCG and *M. smegmatis pknG* TaqMan primer/probe sets were constructed from serial dilutions of known quantities of *in vitro* synthesized transcripts. Threshold cycle (*C*t) values were plotted against copies of transcripts. C and D. PknG mRNA expression in *M. bovis* BCG and *M. smegmatis* per pg of total RNA (C) and per pg of total protein (D). Copies pg^−1^ total RNA were calculated from the standard curves shown in (B). These values were subsequently corrected by the RNA : protein ratio (see *Experimental procedures*) to calculate the number of PknG mRNA copies pg^−1^ total protein. Results are mean values (±SD) from three RNA isolations. *M. smegmatis* PknG mRNA levels were not significantly different from *M. bovis* BCG PknG mRNA levels (*P* > 0.05).

The extremely low levels of PknG from *M. smegmatis* could be explained by inefficient transcription of the *pknG* gene. To analyse *pknG* transcription, total RNA was isolated from *M. smegmatis* or *M. bovis* BCG and analysed for the presence of *pknG* transcripts by RT-PCR (Fig. 7A). Strikingly, a specific band could be amplified by RT-PCR using *M. smegmatis* as well as *M. bovis* BCG RNA with *pknG*-specific primers (only after reverse transcription), indicating the presence of *pknG* RNA in *M. smegmatis.* Sequencing confirmed that this product was the anticipated amplified fragment of the PknG transcripts (not shown).

To accurately measure the levels of PknG transcripts in *M. bovis* BCG and *M. smegmatis*, quantitative real-time RT-PCR was carried out using *in vitro* synthesized PknG^BCG^ and PknG^*smeg*^ transcripts as standards (Fig. 7B). Results are expressed relative to total cellular RNA or protein. This allowed comparison of the differences in *pknG* transcription relative to stable RNA (rRNA, tRNA) or mRNA translation products in these cells (see *Experimental procedures*). When normalized to stable RNA, *M. bovis* BCG had a four times higher level of *pknG* transcript (Fig. 7C). When normalized to cellular protein however, *pknG* mRNA levels in *M. smegmatis* were not significantly different (*P* = 0.23) compared with *M. bovis* BCG (Fig. 7D). We conclude that the sharply decreased levels of PknG in *M. smegmatis* as analysed by immunoblotting or MS analyses are mainly due to a reduced translational efficiency.

### Regulation of *pknG* translation

To address whether the severe difference in the translational efficiency of PknG between BCG and *M. smegmatis* could be due to regulatory elements upstream of the two *pknG* genes, fusion constructs were produced. The ribosome binding site (RBS) in the *hsp60* promoter of pMV361*-pknG*^BCG^ and pMV361*-pknG*^*smeg*^ was replaced by the upstream region of the corresponding *pknG* genes. Since *pknG* has been proposed to be the last gene of an operon of three genes, the two upstream genes of *pknG* were introduced (Fig. 8A; see also *Experimental procedures*). In addition, the upstream regions were exchanged between the constructs; the upstream region of *M. smegmatis pknG* was cloned upstream of BCG *pknG* and visa versa. The constructs were subsequently introduced in the ‘PknG minus’ background of *M. bovis* BCGΔ*pknG* and PknG expression was analysed via Western blotting and RT-PCR (Fig. 8B and C).

Exponentially growing cultures of the *M. bovis* BCG transformants were analysed by Western blotting using an antiserum raised against PknG^BCG^ (Fig. 8B). PknG^BCG^ translationally controlled by its own upstream region in pMV361 was clearly expressed in *M. bovis* BCGΔ*pknG* (Fig. 8B, lane 9), and the product migrated similarly to PknG from *M. bovis* BCG expressed endogenously (Fig. 8B, lane 7). In contrast, no PknG signal was detectable after introduction of the construct containing *M. smegmatis pknG* with its own upstream region in *M. bovis* BCGΔ*pknG* (lane 10). This shows that the low translation of PknG^*smeg*^ is not related to any *M. smegmatis*-specific factors. However, introduction of the construct, in which the *M. smegmatis* gene was fused to the *M. bovis* BCG upstream region, resulted in clear expression of *M. smegmatis* PknG (lane 11), while after introduction of the construct containing BCG *pknG* with the *M. smegmatis* upstream region no PknG signal could be detected (lane 12). From this we conclude that the upstream region of *M. smegmatis pknG* is solely responsible for the virtual lack of expression of the *M. smegmatis* gene.

To verify that the lack of expression of the *pknG* genes fused to the *M. smegmatis* upstream region occurred on a translational level, total RNA was isolated of *M. bovis* BCG wt and the various BCG transformants and subsequently analysed for the presence of *pknG* transcripts by RT-PCR (Fig. 8C). A specific band could be amplified by RT-PCR only in the presence of the reverse trancriptase (RT) using RNA from *M. bovis* BCG wt and all *M. bovis* BCGΔ*pknG* tranformants, except *M. bovis* BCGΔ*pknG* that was transformed with the empty vector (lane 5). We can therefore conclude that the lack of PknG expression in *M. bovis* BCGΔ*pknG* transformed with *pknG* genes fused to the upstream region of *M. smegmatis* is caused by a expression failure on a translational level.

## Discussion

Pathogenic mycobacteria survive within macrophages by preventing fusion of mycobacterial phagosomes with lysosomes (Armstrong and Hart, 1971; Russell, 2001; Houben *et al*., 2006). A crucial molecule involved in the modulation of phagosome lysosome fusion is PknG, a eukaryotic-like mycobacterial serine/threonine kinase (Walburger *et al*., 2004). Notably, while PknG functions as an important virulence factor in pathogenic mycobacteria, a *pknG* gene is also found in a saprophytic species, *M. smegmatis*. Here we show that when expressed from expression vectors, *M. smegmatis pknG* encodes an enzymatically active serine/threonine kinase that provides normal biological activity in the survival of mycobacteria inside macrophages. However, virtually no translation product of endogenous *pknG* was detected either when *M. smegmatis* was grown *in vitro* or within macrophages under the conditions examined here. Quantitative transcriptional assays showed that the sharply reduced expression in *M. smegmatis* could not be attributed to mRNA levels, showing that PknG expression is blocked on a post-transcriptional level in this non-pathogenic mycobacterium.

Replacement of the upstream regions of *M. smegmatis* and *M. bovis* BCG *pknG* by vector-encoded transcriptional and translational expression sequences resulted in similar levels of PknG^*smeg*^ and PknG^BCG^ expression in *M. smegmatis*. This led to the conclusion that the differences in accumulation of *pknG* gene products could be caused neither by post-translational mechanisms such as host-specific proteases, nor by the codon usage within the *pknG* genes. The latter is in agreement with the observation that the codon biases of *M. smegmatis* and *M. bovis* BCG *pknG*, represented by their codon adaptation indices (de Miranda *et al*., 2000), do not significantly differ (not shown). However, replacing the vector-encoded translational expression sequences by their own or each others upstream regions of the *pknG* genes, while keeping the transcriptional sequences of the vector intact, clearly showed that elements in the upstream regions of the genes cause the differences in translation efficiencies. A typical upstream translational regulatory element is the ribosome binding site and it is a possibility that the ribosome binding site of *M. smegmatis pknG* is very inefficient. Alternatively, recognition of the upstream region by regulatory RNA, proteins or metabolites might cause the differences in translational efficiencies between mycobacterial species.

**Fig. 9:**
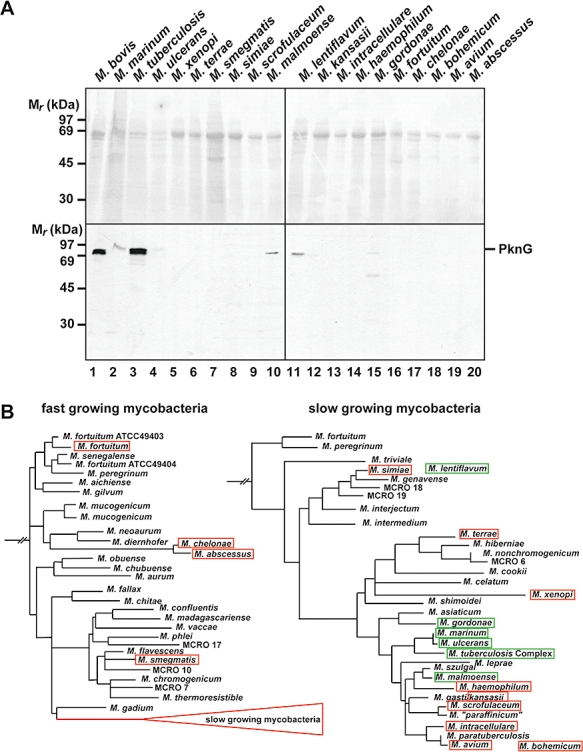
Analysis of *pknG* expression in a wide range of mycobacterial species. A. Lysates of various mycobacterial species were separated on a 10% SDS-PAGE gel and immunoblotted using anti-PknG antiserum (bottom). The total protein pattern was analysed by Ponceau Red staining (top). For sources and growth conditions of the mycobacterial species see Table S2. B. Phylogenic tree of mycobacteria based on 16S rRNA gene sequences. Species that show a PknG signal in (A) are boxed in green, the ones that do not show expression are boxed in red. Tree adapted from Springer *et al*. (1996).

Western blot analysis for the expression of PknG in a wide range of mycobacterial species grown under similar growth conditions revealed that only some of the slow-growing pathogenic species, mainly the members of the *M. tuberculosis* complex and species closely related to the *M. tuberculosis* complex, expresses PknG, while none of the analysed non-pathogenic, fast-growing species showed PknG expression (Fig. 9). Notably, all mycobacterial genomes, of which sequence data are available at this moment (from both pathogenic and non-pathogenic species), contain a *pknG* gene. Therefore, *pknG* might be conserved in all mycobacterial species, which implies that not only in *M. smegmatis*, but in more mycobacterial species PknG expression is suppressed.

The availability of a growing number of mycobacterial genome sequences allows comparisons that may help to identify genes corresponding to strain-specific pathogenicity functions (Cole *et al*., 2001). Our studies suggest the existence of another subset of genes whose relative levels of expression, and not their presence or absence, defines virulence in mycobacterium. It is important to recognize that the changes on the level of expression that determine *pknG* activity in different species might be achieved by the evolution of regulatory systems that target subsets of mycobacterial virulence genes. Identification and analysis of these regulatory systems may provide new strategies to combat important mycobacterial diseases such as tuberculosis and leprosy.

## Experimental procedures

### Cell lines, bacterial strains and antibodies

*Mycobacterium bovis* BCG Pasteur was provided by the Institut Pasteur (Paris, France) and *M. smegmatis* mc^2^155 was from ATCC (ATCC 700084). Disruption of *pknG* in the *M. bovis* BCG Pasteur was carried out as described (Walburger *et al*., 2004). Mycobacteria were grown in 7H9 mycobacterial medium supplemented with 10% OADC Middlebrook supplement (Difco Laboratories). Bone marrow-derived macrophage isolation from C57/BL6 mice, anti-LAMP1 monoclonal antibodies, secondary reagents and the PknG inhibitor AX20017 have been described (Ferrari *et al*., 1999; Gatfield and Pieters, 2000; Walburger *et al*., 2004). Antibodies against PknG were raised by Eurogentec in New Zealand white rabbits against His-tagged recombinant PknG that was expressed in *E. coli* and purified as described (Walburger *et al*., 2004). PknG-specific IgGs were further purified using a HiTrap Protein A column, followed by a HiTrap NHS-activated column coupled with His-tagged recombinant PknG (both supplied by GE Healthcare Bio-Sciences). Polyclonal rabbit antibodies against *M. bovis* were purchased from Dako Corporation.

### Cloning of PknG expression vectors

Anchored primers (EcoRI–NdeI for the 5′ primer and NdeI–HindIII for the 3′ primer) were used to amplify *pknG* from *M. smegmatis* genomic DNA by PCR, cloned in pGEM-T-Easy (Promega) and sequenced (Applied Biosystems). After sequencing, the genes were cloned as EcoRI–HindIII fragments in pMV361 (Stover *et al*., 1991) and in pBAD/His B (Invitrogen Life Technologies). The resulting plasmids were designated pMV361*-pknG*^*smeg*^ and pBad-PknG^*smeg*^. An anchored 5′ primer (AclI-BCG0451c.fw containing an AclI site; see Table S1) and a 3′ primer recognizing a sequence downstream of the AclI site in *M. bovis* BCG *pknG* (BCG-Pk-EcoRV.rev) were used to amplify *pknG* together with 2345 bp upstream region (containing BCG0431c and *glnH* including 40 bp upstream of BCG0431c) from *M. bovis* BCG genomic DNA by PCR and cloned AclI–AclI in pMV361*-pknG*^BCG^ (Walburger *et al*., 2004), designated pMV361-BCG-*pknG*^BCG^ (see also Fig. 8A). Since the AclI site in the *hsp60* promoter region of pMV361 is located upstream of the ribosome binding site, but downstream of the transcription start (Stover *et al*., 1991), only the transcription start of *hsp60* is left in this construct. Similarly, an anchored 5′ primer with an AclI site (AclI-MSMEG_0788.fw) was used together with a 3′ primer recognizing the XmnI site in *M. smegmatis pknG* (Smeg-XmnI.rev) to amplify *M. smegmatis pknG* together with the 2423 bp upstream region (containing MSMEG_0788, MSMEG_0787 and 40 bp upstream of MSMEG_0788) from *M. smegmatis* genomic DNA and cloned AclI–XmnI into pMV361*-pknG*^*smeg*^ (this study), resulting in pMV361-*smeg-pknG*^*smeg*^. In addition, the upstream regions of the two genes were exchanged by a nested PCR approach (by using the 5′ anchor primers, the 3′ AclI or XmnI primer and internal overlapping primers; for primer sequences see Table S1), resulting in pMV361-BCG-*pknG*^*smeg*^ for *pknG*^smeg^ with the upstream region of *pknG*^BCG^ and pMV361-*smeg-pknG*^BCG^ for *pknG*^BCG^ together with the upstream region of *pknG*^smeg^. All constructs were sequenced (Applied Biosystems) and electroporated into *M. smegmatis* mc^2^155 and/or *M. bovis* BCGΔ*pknG* Pasteur (Parish and Stoker, 1998).

### Expression and purification of *M. smegmatis* PknG

For expression and purification of *M. smegmatis* PknG, *E. coli* strain BL21 was transformed with pBAD-PknG^*smeg*^. Transformants were grown in LB medium containing 100 μg ml^−1^ ampicillin and 0.2% glucose at 37°C until the OD_600_ reached 0.45. Bacteria were pelleted (4200 *g* for 10 min) and re-suspended in LB medium containing 100 μg ml^−1^ ampicillin and 0.2% arabinose. Bacteria were grown for another 4 h at 37°C and harvested (4200 *g* for 10 min at 4°C). (His)_6_-PknG^*smeg*^ was purified via a Ni^2+^-loaded HisTrap chelating column from GE Healthcare Bio-Sciences using an imidazole gradient as described (Walburger *et al*., 2004).

### Biochemical methods

Homogenization of mycobacterial lysates was performed and kinase activity of purified kinase was measured as described (Walburger *et al*., 2004).

### RT-PCR and qRT-PCR

Isolation of RNA samples from *M. bovis* BCG and *M. smegmatis*, DNase treatments, cDNA synthesis and the amplification of *pknG* cDNA has been described (Nguyen *et al*., 2005). The protein concentration of the mycobacterial lysates and the amount of RNA isolated from these lysates were determined using a Bradford protein assay and the NanoDrop spectrophotometer respectively. Protein and RNA concentrations were additionally verified on SDS-PAGE and agarose gels respectively. Amplification of *pknG* cDNA from *M. bovis* BCG and *M. smegmatis* was carried out using the same primers (RT-PknG.fw and RT-PknG.rev in Table S1). As a control, transcription of the 16S rRNA gene (16S-RNA.fw and 16S-RNA.rev in Table S1) was analysed as well.

The same RNA samples were used for quantitative RT-PCR. In addition, *M. bovis* BCG and *M. smegmatis* PknG transcripts were synthesized from HindIII linearized pET15b-PknG^BCG^ (Walburger *et al*., 2004) and pGEM-T-Easy-PknG^*smeg*^ using the T7 RiboMAX express large-scale RNA production system (Promega) and used as standards. Quantitative, real-time, one-step RT-PCR was carried out using the Applied Biosystems 7500 Sequence Detection Systems (SDS). Sets of primer pairs and a TaqMAN probe (Table S1) were designed and qRT-PCR was performed using the QuantiTect probe RT-PCR kit (QIAGEN). Fluorescence data were analysed using the Applied Biosystems Sequence Detection software version 1.2.3. Each reaction was run in duplicates.

### Sample preparation for mass spectrometric analysis

The region between 65 and 100 kDa of each entire lane was excised followed by in-gel trypsin digestion as described (Granvogl *et al*., 2006).

### Nano-LC-IT-SIM-MS analysis

The resulting peptides from each gel piece were analysed by liquid chromatography (LC) coupled to a LTQ ion trap mass spectrometer (Thermo Electronics) equipped with a nano-LC electrospray ionization source. Peptides were dissolved in buffer A (1% formic acid, 0.5% acetic acid, 0.012% heptafluorobutyric acid) and concentrated and desalted online on a C18 PepMap 100 micro precolumn (5 μm particle size, 300 μm × 1 mm; Dionex Corporation) that was coupled to a self-packed (7 cm, 3 μm; ProntoSil C18-ACE-EPS, Bischoff Chromatography) and pulled (P-2000 laser puller, Sutter Instrument) fused silica capillary (100 μm inner diameter × 365 μm outer diameter). The chromatographic separation was then performed by a 100 min non-linear gradient from 5–55% buffer B (80% acetonitrile/0.5% acetic Acid/0.012% HFBA) with a constant flow rate of 0.20 μl min^−1^. The mass spectrometric data acquisition was performed with a survey scan (450–1500 m/z) followed by five selected ion monitoring scans (SIM). The selected masses correspond to the following peptides: LGGGLVEIPR [MH2^+^ 505.8], LIDLGAVSR [MH2^+^ 472.28], TGPTVATDIYTVGR [MH2^+^ 725.88], LTSAVTLLSGR [MH2^+^ 559.33], TLAALTLDLPTR [MH2^+^ 642.88] and ALLDLGDVAK [MH2^+^ 507.28]. The collision energy was set to 35%.

### Database searching for protein identification

SEQUEST (Bioworks, Thermo Electron Corporation) was used to search the SWISSPROT database (UniprotKB/Swiss-Prot) supplemented with the corresponding *pknG* sequence of *M. smegmatis* for peptide sequence and protein identification. Search parameters included differential mass modification to methionine due to possible oxidation and static mass modification to cysteine due to alkylation by iodoacetamide. Furthermore, one missed cleavage of trypsin was accepted.

### Statistical analysis

Data were statistically analysed using the Student's *t*-test and differences were considered significant if the *P*-value was < 0.05.
