# Supplementary material for: Differential expression of a virulence factor in pathogenic and non-pathogenic mycobacteria
Source: Mol Microbiol. 2009 Feb 19;72(1):41–52. doi: 10.1111/j.1365-2958.2009.06612.x (PMC2784876; doi:10.1111/j.1365-2958.2009.06612.x)
Supplement: Supplementary file 1 [file mmi0072-0041-SD1.pdf]

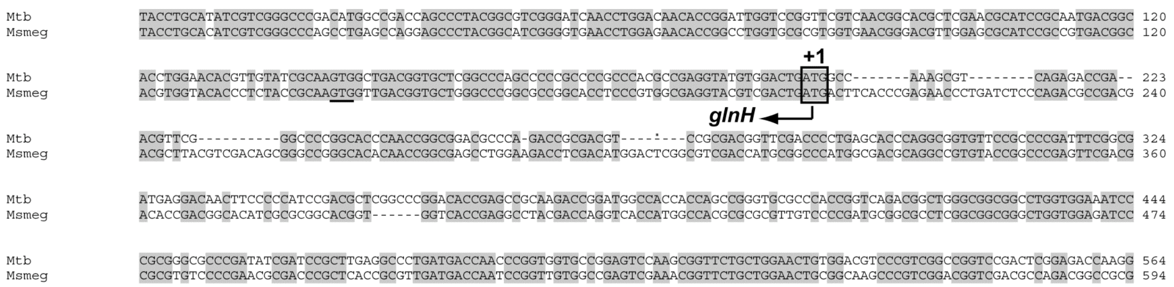

Figure S1. Investigation of the translational start of *M. smegmatis pknG*. Alignment of the first 328 and 388 nucleotides of *pknG* from *M. tuberculosis* (Mtb) and *M. smegmatis* (Msmeg), respectively, including the upstream 200 nucleotides. The limits of gene upstream of *pknG*, *glnH*, is indicated. Identical residues are shown in grey. The translational start codon of *M. smegmatis pknG* was annotated on the TIGR Comprehensive Microbial Resource (CMR) home page at a GTG (underlined) further upstream of the kinase domain compared to the other *pknG* homologues (see Figure 1). However, alignment of the *M. smegmatis* and *M. tuberculosis pknG* including their upstream nucleotides revealed not only that the upstream regions are highly conserved as well, but also that an ATG downstream of the annotated start codon in the *M. smegmatis* sequence aligned to the start codon of *M. tuberculosis pknG* (both are framed). Since *M. tuberculosis* PknG with this start codon is an active kinase (Walburger et al., 2004) and rescues *M. bovis* BCG Δ*pknG* from lysosomal delivery when expressed in *trans* (Houben et al., 2006), we propose this ATG is the most probable start codon of *M. smegmatis pknG*. *M. smegmatis pknG* with this start codon was cloned into pMV361 to obtain pMV-PknG^{smeg}.

Table S1. List of primers and probe used for cloning of the constructs analyzed in Figure 8 and for the (q)RT-PCR reactions in Figure 7 and 8.

Primer name	Sequence (5' to 3')
AclI-BCG0451c.fw	GTGCTAAGAATAACGTTAGTTTCGTTGCGTTAG CCGAA
BCG-Pk-EcoRV.rev	CCTCAAGCGGATCGATATCG
AclI-MSMEG_0788.fw	GTGCTAAGAATAACGTTAGAGCCGTAAGCATCG AAAC
Smeg-Pk-XmnI.rev	CAGTTCCAGCAGAACCGTTTC
Smeg-Pk_BCG-UTR.rev	GGTGAAGTCATCAGTCCACATACCTCGG
BCG-Pk_Smeg-UTR.rev	GCTTTGGCCATCAGTCGACGTACCTCG
BCG-UTR_Smeg-Pk.fw	TATGTGGACTGATGACTTCACCCGAGAACCC
Smeg-UTR_BCG-Pk.fw	ACGTCGACTGATGGCCAAAGCGTCAGAGAC
RT-PknG.fw	GCCACCGACATCTACACCGT
RT-PknG.rev	GGTGTGCGCCACCAGCAG
16S-RNA.fw	ACGAACAACGCGACAAACC
16S-RNA.rev	CCAGCAGCCGCGGTAA
qRT-BCG.fw	CGGCCAGTACGAGGTCAAAG
qRT-BCG.rev	GCGGTCGAGAGCGAGGTAG
qRT-smeg.fw	CGACCAGTACGAGATCAAGGGT
qRT-smeg.rev	TCTTGTCGAACGCCAGGTACA
PknG-probe	6FAM-CCGCCGTGCGCGAT-MGBNFQ

Table S2. Sources and growth conditions of the various mycobacterial species analyzed for PknG expression in Figure 9.

Mycobacterial species	Source	Medium	Temperature
M. abscessus	ATCC 19977	mod. LJ*	35°C
M. africanum	Swiss NCM ***	mod. LJ	35°C
M. avium subsp. avium	MAC101	mod. LJ	35°C
M. bohemicum	Clinical isolate	mod. LJ	35°C
M. bovis	ATCC 19210	mod. LJ	35°C
M. bovis biovar. BCG	ATCC 35734	mod. LJ	35°C
M. bovis subsp. caprae	Clinical isolate	mod. LJ	35°C
M. chelonae	DSM 43804	mod. LJ	35°C
M. fortuitum	ATCC 49403	mod. LJ	35°C
M. gordonae	Pasteur 14021.001	mod. LJ	35°C
M. haemophilum	ATCC 29548	MB 7H10**	28°C
M. intracellulare	Clinical isolate	mod. LJ	35°C
M. kansasii	NCTC 10268	mod. LJ	35°C
M. lentiflavum	Clinical isolate	mod. LJ	35°C
M. malmoense	NCTC 11298	mod. LJ	35°C
M. marinum	ATCC 927	mod. LJ	28°C
M. scrofulaceum	Pasteur 14022.0031	mod. LJ	35°C
M. simiae	Clinical isolate	mod. LJ	35°C
M. smegmatis	Pasteur 14133.0001	mod. LJ	35°C
M. terrae	Clinical isolate	mod. LJ	35°C
M. tuberculosis H37Rv	Pasteur 14001.0001	mod. LJ	35°C
M. xenopi	Clinical isolate	mod. LJ	35°C

* modified Löwenstein-Jensen
** Middlebrook with 10% OADC-Enrichment supplement with 0.4% Ferrum-Ammonium-Citrat
***Swiss NCM: Swiss National Centre for Mycobacteria, Zurich.
